# Transamidation Down-Regulates Intestinal Immunity of Recombinant α-Gliadin in HLA-DQ8 Transgenic Mice

**DOI:** 10.3390/ijms22137019

**Published:** 2021-06-29

**Authors:** Stefano Rossi, Deborah Giordano, Maria Fiorella Mazzeo, Francesco Maurano, Diomira Luongo, Angelo Facchiano, Rosa Anna Siciliano, Mauro Rossi

**Affiliations:** 1 Immunobiology Unit, CNR, Institute of Food Sciences, 83100 Avellino, Italy; s.rossi.uni@gmail.com (S.R.); maurano@isa.cnr.it (F.M.); diomira.luongo@isa.cnr.it (D.L.); 2 Bioinformatics and Computational Biology Unit, CNR, Institute of Food Sciences, 83100 Avellino, Italy; deborah.giordano@isa.cnr.it (D.G.); angelo.facchiano@isa.cnr.it (A.F.); 3 Proteomics and Biomolecular Mass Spectrometry Center, CNR, Institute of Food Sciences, 83100 Avellino, Italy; fmazzeo@isa.cnr.it (M.F.M.); rosa.siciliano@isa.cnr.it (R.A.S.)

**Keywords:** celiac disease, HLA-DQ8, immunomodulation, in silico analysis, mass spectrometry, recombinant alpha gliadin, small intestine, transamidation, microbial transglutaminase

## Abstract

Enzymatic transamidation of gliadins by microbial transglutaminase (mTG) inhibits interferon-γ (IFN-γ) secretion by intestinal T cell lines in patients with celiac disease (CD). To gain insight into the cellular mechanisms underlying the down-regulatory effects of transamidation, we tested a single recombinant α-gliadin (r-gliadin) harbouring two immunodominant peptides, p13 (aa. 120–139) and p23 (aa. 220–239), in HLA-DQ8 transgenic mice, a model of gluten sensitivity. Mice were intranasally immunised with r-gliadin or r-gliadin transamidated by mTG (K-r-gliadin) along with cholera toxin, and the response of mesenteric lymph node cells was analysed by cytokine multiplex assay. An in vitro challenge with r-gliadin was characterised by secretion of specific cytokines featuring both innate immunity and the Th1/Th2/Th17 pattern of the adaptive response. Notably, transamidation specifically down-regulated the Th1 response. Structural studies performed on K-r-gliadin confirmed that specific glutamine residues in p13 and p23, previously found to be deamidated by tissue transglutaminase, were also transamidated by mTG. In silico analysis, simulating p13 and p23 peptide binding to HLA-DQ8 showed that these glutamines, in the form of glutamate, could interact by means of salt bridges with peculiar amino acids of the alpha chain of HLA-DQ8, suggesting that their transamidation may influence the HLA-restricted recognition of these peptides. Thus, the structural findings provided a rationale to explain the down-regulation of the r-gliadin-specific Th1 response following transamidation.

## 1. Introduction

Celiac disease (CD) is a common food-sensitive enteropathy in humans caused by absence of oral tolerance to wheat gluten. Almost all patients with CD express the MHC class II molecules HLA-DQ2.5, HLA-DQ8, or HLA-DQ2.2, with HLA-DQ2.5 being the major genetic risk factor [1]. This suggests the dominant role of adaptive immunity in the pathogenesis of CD. In particular, specific gliadin peptides could bind to DQ2 and DQ8 heterodimers on the surface of antigen presenting cells (APCs), thus inducing CD4^+^ T cell activation in the intestinal lamina propria [2]. However, a preliminary deamidation step catalysed by tissue transglutaminase (tTG) appears crucial [3], as the negative charges introduced in the gliadin molecules by this reaction significantly increase the binding affinity of peptides to DQ2 [4,5,6] and DQ8 [7] heterodimers. Interestingly, peptide analogues of the immunodominant 33-mer peptide [8] efficiently inhibited antigen presentation in a dose-dependent manner [9], thus providing archetypal examples of antigen manipulation for the management of CD. We previously showed that enzymatic transamidation of gliadin by microbial transglutaminase (mTG) was effective at blocking IFN-γ production by intestinal T cells in patients with CD [10]. mTG is a transamidase of the endo-γ-glutamine:ε-lysine transferase type [11]. Unlike tTG, mTG is a calcium-independent, low molecular weight protein, which has several advantages in food industrial applications [12]. Similar to tTG, mTG catalyses a transamidation reaction that leads to the formation of an isopeptide bond. Specifically, the carboxamide group of a peptide-bound glutamine residue (Q) is the acyl donor and an appropriate primary amine, such as the ε-amino group of a lysine residue, is the acyl acceptor [13]. Importantly, structural analyses of gliadins extracted from wheat flour treated with mTG performed by applying methodologies based on tandem mass spectrometric experiments (i.e., nano-HPLC-ESI-MS/MS) identified different transamidated forms of celiacogenic peptides and confirmed that mTG had a similar site-specificity of tTG [14]. This indicated that transamidation reactions could effectively prevent the formation of immunodominant gliadin peptides. In silico analysis of peptide-DQ binding showed that transamidation caused a decrease in the energy of interaction due to the loss of H-bonds and electrostatic energy [10], thus providing a structural basis for our immunological findings. In HLA-DQ8 transgenic (DQ8) mice, the enzymatic modification of gliadin was associated with an alteration of the gliadin-inducible IL-10/IFN-γ ratio in spleen cells, indicative of reversal of the phenotype from inflammatory to anti-inflammatory [15]. In the same mouse model, we also showed the ability of transamidated gliadin to prevent the inflammatory phenotype triggered by native gliadin [16]. This property was intrinsically associated with specific components of the α-gliadin fraction. In the present study, we further evaluated the anti-inflammatory properties of transamidated gliadin by analysing the intestinal response toward a single recombinant α-gliadin in DQ8 mice. We confirmed down-regulation of the inflammatory phenotype consequent to transamidation that could reduce the peptide binding affinity for the DQ8 pocket. Finally, by molecular simulations, we proposed a model of interaction between two immunodominant peptides and HLA-DQ8, with an interpretation of their specific recognition in patients with CD.

## 2. Results

### 2.1. Biochemical Assessment of Transamidated Recombinant Alpha-Gliadin

**Figure 1:**
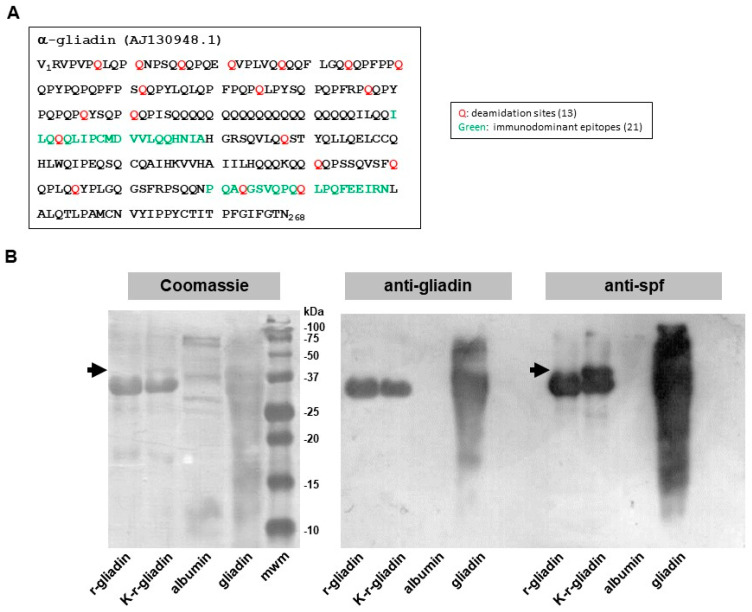
Biochemical characterization of transamidated recombinant α-gliadin. (**A**) Amino acid sequence of the α-gliadin AJ130948.1. The glutamine deamidation sites and the two immunodominant peptides are highlighted in red and green, respectively. (**B**) SDS-PAGE (**left panel**) and Western blot analysis of recombinant α-gliadin (r-gliadin) and its transamidated form (K-r-gliadin) using anti-native gliadin (**middle panel**) or anti-spf mouse polyclonal antibody probes (**right panel**). The arrows indicate the additional band in K-r-gliadin sample.

Gliadin is a mixture of various related proteins, thus making it problematic to associate a specific biological activity with a particular protein. To address this issue, we used a recombinant α-gliadin (r-gliadin) whose primary sequence (Figure 1A) was highly conserved among α-gliadins retrieved from Swiss-Prot [17]. r-gliadin was then subjected to enzymatic treatment with mTG and lysine ethyl ester to produce a transamidated r-gliadin (K-r-gliadin). Coomassie blue staining after SDS-PAGE revealed that r-gliadin was almost 90% pure (Figure 1B, left panel). K-r-gliadin resolved an additional discrete band with higher molecular weight (mw). As expected, the resolution of wheat gliadin was not clear enough to detect the different fractions separately [18]. In particular, a main band resulted encompassing different α and β subunits, as normally found for gliadins isolated from bread wheat genotypes [18]. Western blot analysis showed that anti-gliadin polyclonal antibodies cross-reacted with r-gliadin and K-r-gliadin (Figure 1B, right panel). Interestingly, probing with polyclonal antibodies raised against transamidated gliadin (anti-spf) [14] confirmed the presence of a new cross-reacting band with higher mw.

### 2.2. Analysis of Transamidated Gliadin in DQ8 tg Mice

**Figure 2:**
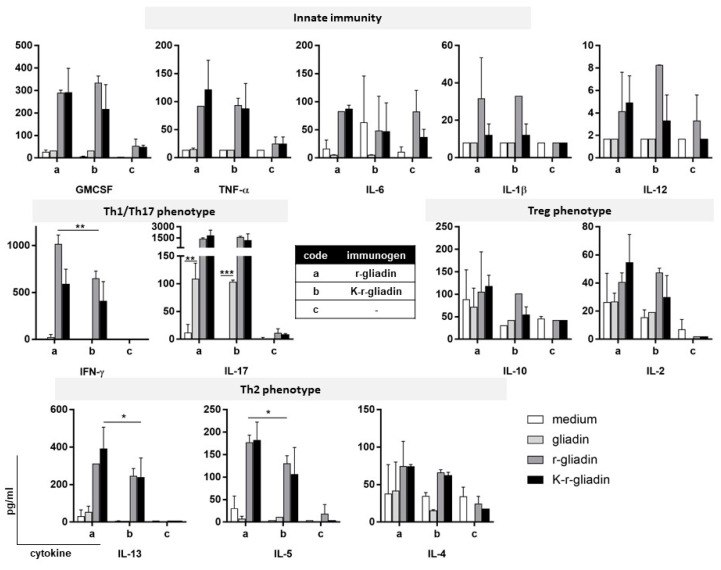
Intestinal cytokine secretion of r-gliadin in DQ8 tg mice. Mice were intranasally administered r-gliadin or K-r-gliadin along with CT. Antigen-specific cytokine expression was assessed by culturing MLN cells from immunized mice for 72 h with the different antigens (*n* = 3). Supernatants were then assayed by multiplex assay. Values are presented as mean ± SD. The results are representative of three independent experiments. *, *p* < 0.05; **, *p* < 0.01; and ***, *p* < 0.001.

To gain insight into the mechanisms underlying the intestinal immune response triggered by transamidated gliadin, we mucosally immunized DQ8 tg mice on a gluten-free diet by nasal administration of r-gliadin or K-r-gliadin plus cholera toxin (CT). Mesenteric lymph node (MLN) cells were then challenged in vitro with both forms of recombinant protein or wheat gliadin. Culture supernatants were analysed for simultaneously monitoring the quantitative expression of 12 cytokines in a multiplex analysis. Nasal sensitization induced both innate and adaptive antigen-specific immune responses in MLNs (Figure 2). Among five examined cytokines of innate immunity (GMCSF, IL-6, IL-1b, IL-12 and TNF-α), we found that both r-gliadin and K-r-gliadin specifically stimulated secretion of GMCSF and TNF-α at comparable levels. On the other hand, only MLN cells isolated from mice treated with r-gliadin expressed IL-6. The analysis of adaptive immunity showed that r-gliadin-specific MLN cells produced lower amounts of IFN-γ when challenged with K-r-gliadin than with r-gliadin, as previously described for transamidated wheat gliadins [10,15,16]. In addition, stimulated MLN cells also produced Th2-like cytokines, IL-5 and IL-13, but not IL-4. However, differently from IFN-γ, transamidation did not reduce IL-5 and IL-13 secretions. The analysis of MLN cells isolated from mice immunized with K-r-gliadin showed that preventive transamidation decreased the intensity of the overall adaptive response but not its phenotype, as lower secretions of IFN-γ, IL-5, and IL-13 were detected following cell stimulation with both antigens. Notably, we found that IL-17 production was very peculiar. In particular, this response was characterised by a strong antigen-specific, transamidation-independent secretion. Furthermore, in vitro challenge with wheat gliadins also induced some levels of IL-17 secretion in sensitized MLN cells. Finally, both forms of the recombinant antigen were unable to stimulate the regulatory cytokines IL-10 and IL-2 in any of the examined challenges.

### 2.3. Mass Spectrometry Analysis of Immunodominant Peptides of r-Gliadin

**Figure 3:**
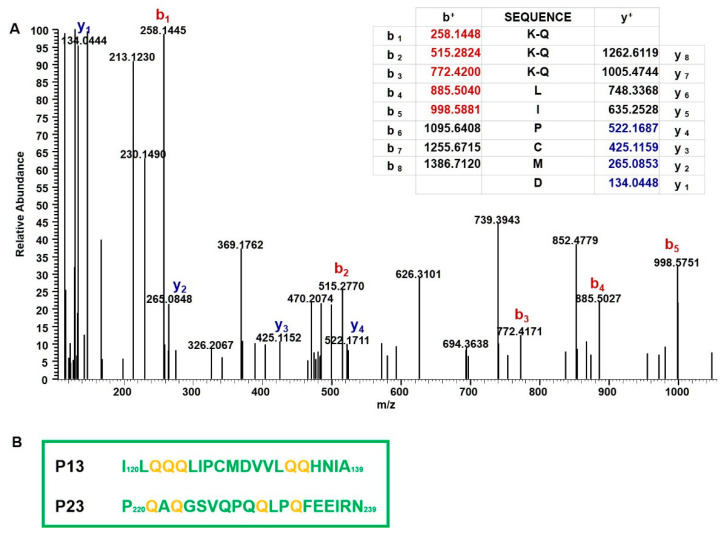
Identification of Q residues transamidated by mTG. (**A**) MS/MS spectrum of the peptide QQQLIPCMD (peptide 122–130 of the K-r-gliadin) containing three modified Q residues (precursor ion at *m*/*z* 507.257). The table included in the figure reports the theoretical b and y fragment ions for the fully modified peptide. Signals present in the MS/MS spectrum matched to b ions (highlighted in red) and y ions (highlighted in blue). K-Q refers to transamidated glutamine residues. (**B**) Amino acid sequence of p13 and p23. The glutamine residues transamidated by mTG sites are highlighted in yellow.

We previously showed that the intestinal Th1 response in DQ8 mice sensitized with r-gliadin was essentially restricted to two 20-mer peptides encompassing the amino acidic sequences 120–139 (p13) and 220–239 (p23) (Figure 1A) [17]. We also found that glutamine residues of celiacogenic peptides reported to be deamidated by tTG were also transamidated by mTG [14]. To confirm that the herein reported down-regulation of IFN-γ could be associated with transamidation of specific Q residues of p13 and p23, structural studies were carried out. We analysed a peptide mixture obtained from the elastase digestion of K-r-gliadin by using nano-high pressure liquid chromatography-electrospray ionization-tandem mass spectrometric methodologies (nano-HPLC-ESI-MS/MS). These analyses led to identify Q residues present in p13 and p23 mainly transamidated by mTG and modified by the addiction of K instead of K-ethyl ester, which is probably due to a hydrolysis reaction occurring under our experimental conditions. The analysis of the peptide QQQLIPCMD (peptide 122–130 of the K-r-gliadin), encompassed in p13, showed that the three Q residues were all modified (Q122, Q123, and Q124). In fact, the MS/MS spectrum obtained from the precursor ion at *m*/*z* 507.257 (a triply charged ion) was generated from this peptide (MW 1074.484 Da) carrying three modified Q residues (MW increase (ΔM) of 129.079 Da for each K addiction). The signal at *m*/*z* 258.144 that originated from the b1 fragment ion K-Q confirmed that Q122 was actually transamidated. Similarly, the b_2_ and b_3_ fragment ions at *m*/*z* 515.277 and 772.417, respectively, indicated the transamidation of Q123 and Q124, as deduced from a ΔM of 257.138 Da between the b_1_–b_2_ and the b_2_–b_3_ fragment ions (Figure 3A). Furthermore, the two Q residues (Q134 and Q135) of the peptide MDVVLQQH (peptide 129–136 of the K-r-gliadin, included in p13) were also modified by mTG, as deduced from the MS/MS spectrum obtained from the triply charged ion at *m*/*z* 415.218 (Figure S1 in Supplementary Materials). As to p23, the MS/MS spectrum of the quadruply charged ion at *m*/*z* 593.558, originated from the peptide SFRPSQQNPQAQGSVQPQ (peptide 212–229) carrying three transamidated Q residues allowed us to define that Q218, Q221 and Q223 were modified, while the Q residues 217, 227 and 229 were not modified by mTG (Figure S2 in Supplementary Materials). Finally, Q230 and Q233 were also found to be transamidated in the peptide QLPQFEEIR (peptide 230–238), as deduced from the MS/MS spectrum obtained from the fragmentation of the triply charged ion at *m*/*z* 473.263 (Figure S3 in Supplementary Materials). In conclusion, it should be underlined that mTG was able to modify all the Q residues previously reported to be deamidated by tTG (Q123 in p13 and Q223 and Q230 in p23) [13], as well as the following residues: Q122, Q124, Q134, and Q135 in p13 and Q221 and Q233 in p23 (Figure 3B).

### 2.4. Ability of Immunodominant Peptides to Interact with HLA-DQ8 Heterodimer

**Figure 4:**
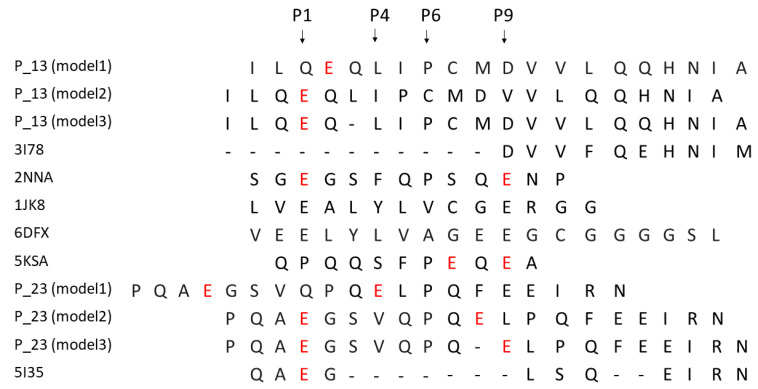
Alignment of p13 and p23 peptide sequences with template peptides, indicated by the PDB code, used for the modelling procedure. Three different options have been defined to obtain models labelled as model 1, model 2 and model 3 for both p13 and p23. The glutamines subjected to deamidation are in red.

**Figure 5:**
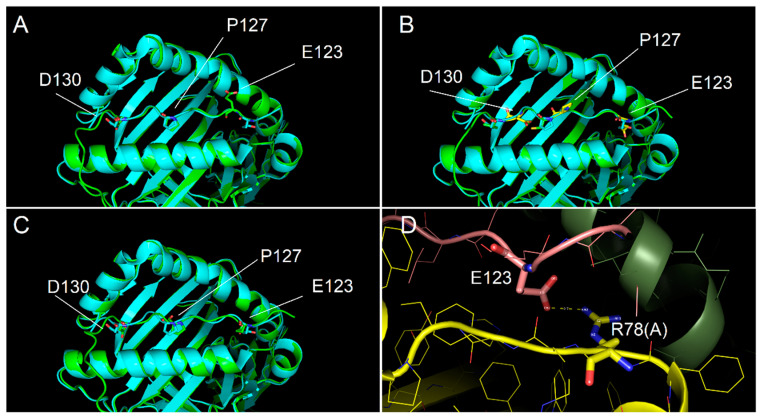
Interaction of p13 with DQ8 binding site. (**A**–**C**) Interaction models obtained with the alignment conditions named model 1, model 2 and model 3, respectively. Cyan and green models overlapped showed the conformity of experimental (PDB code 2NNA) and modelled structures, respectively, of the DQ8 protein shown with backbone ribbon and of experimental peptide and modelled p13. Labels identify key residues of the modelled peptide, differently positioned in the binding groove as for the different alignment used. In model 3, all key residues of p13 are very close to the position of the corresponding residues of the template peptide in the anchor sites. (**D**) Detail of the salt bridge interaction between Arg78 of the DQ8 alpha chain and E123 of p13 observed for model 3.

**Figure 6:**
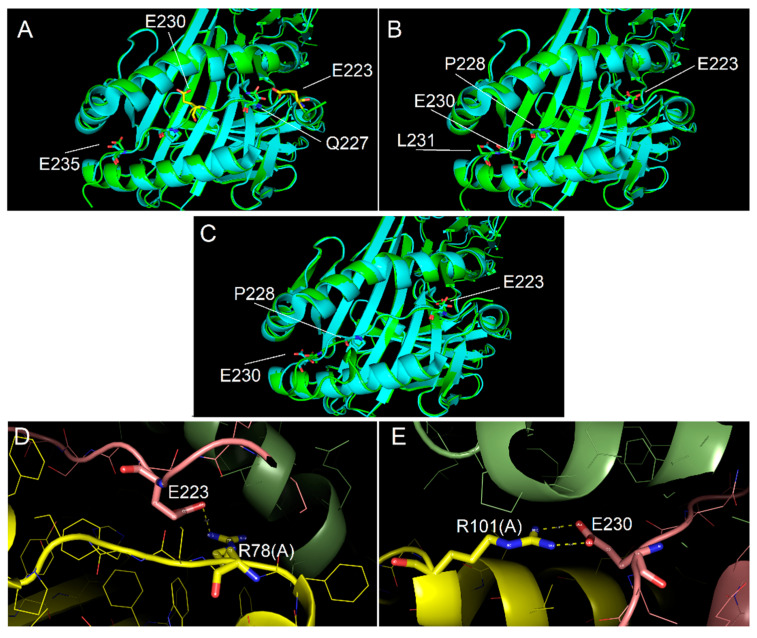
Interaction of p23 with DQ8 binding site. (**A**–**C**) Interaction models obtained with the alignment conditions named model 1, model 2 and model 3, respectively. As for Figure 5, cyan and green models overlapped showed the conformity of experimental (PDB code 2NNA) and modelled structures, respectively, of the DQ8 protein shown with backbone ribbon and of experimental peptide and modelled p23. Labels identify key residues of the modelled peptide, differently positioned in the binding groove as for the different alignment used. In model 3, all key residues of p13 are very close to the position of the corresponding residues of the template peptide in the anchor sites. (**D**) Detail of the salt bridge interactions between Arg78 of the DQ8 alpha chain and E223 of p23 observed for model 3. (**E**) Detail of the salt bridge interactions between Arg101 of the DQ8 alpha chain and E230 of p23 observed for model 3.

In order to model the complexes DQ8-p13 and DQ8-p23, three different alignments of the peptides have been defined, and consequently, three different models for each complex have been obtained. Figure 4 shows the alignment of the p13 and p23 sequences to the peptides present in complex template structures of DQ8. The anchor positions (named P1, P4, P6, and P9) have been described in the literature for their preferred side chains of peptide residues: in P1 and P9, negative or polar residues; in P4, aliphatic/aromatic or neutral residues; and in P6, small aliphatic residues [19]. These conditions are well satisfied by the peptides from the PDB complexes having codes 2NNA, 1JK8, 6DFX, and 5KSA (see Methods for details about the complexes). We aligned the sequences of p13 and p23 with three different options to generate alternative models (named model 1, model 2, and model 3) to fit, at the best, the residues in the anchor positions. The third option, obtained by inserting a gap, made it possible to get negative side chains into the P1 and P9 positions (Figure 4) for both peptides. The negative side chain of glutamate in P1 could be preferred, being the residue in P1 in template peptides from the 2NNA, 1JK8, and 6DFX complexes. In Figure 5 and Figure 6, we show the structure of the three models of p13 and p23, respectively, in the DQ8 binding site. By evaluating the quality of the models, the best result was obtained for model 3 for both peptides. This was an interesting result, because it was obtained following an alignment with a gap along the amino acid sequence (see Discussion for further considerations). As it is shown, model 3 of p13 (Figure 5) and model 3 of p23 (Figure 6) were able to adapt the side chains in the anchor positions similarly to the peptide already present in the template structure. Furthermore, in the case of p13, we noted that glutamate in P1, corresponding to the deamidated form of Q123, may link an arginine of the DQ8 alpha chain by a salt bridge (Figure 5). This amino acid is Arg78 in the UniProt entry P19019, where it is annotated as an object of allele variation (see Discussion). Similarly, in the case of p23, two salt bridges can be formed with amino acids of the same alpha chain. The first one is between the same arginine of the DQ8 alpha chain and the glutamate in P1, which simulates the deamidated Q223. The second salt bridge is between another arginine (i.e., Arg101 of the DQ8 alpha chain (that in UniProt entry P19019 is annotated as an object of variation, too) and the glutamate in P9, which simulates the deamidated Q230. Remarkably, Q123 of p13 and Q223 and Q230 of p23 are targets of transamidation; therefore, their chemical modification precludes the possibility of their involvement in salt bridges, thus opposing the interaction of the peptides with DQ8.

## 3. Discussion

In this study, we showed that transamidation of a recombinant gliadin (r-gliadin) specifically down-regulated the Th1 immune response in the small intestine of sensitized DQ8 transgenic (DQ8) mice. This model, besides expressing a CD-associated HLA molecule, developed a specific gluten sensitivity [20]. In our previous studies, we elicited an intestinal T cell response to r-gliadin in DQ8 mice, identifying two immunodominant peptides, p13 (aa.120–139) and p23 (aa. 220–239) as mainly responsible for the response [17]. Interestingly, tTG treatment neither induced other dominancy nor increased p13 and p23 stimulatory activities. Thus, sensitized DQ8 mice appeared to resemble a condition found in children with CD, which displayed both deamidation-dependent and deamidation-independent responses to gluten peptides [6]. This finding further strengthens the DQ8 mouse as an appropriate model for testing immunomodulatory protocols. Among these protocols, enzymatic transamidation of gliadin appears a promising strategy to exploit. We showed that in vitro challenge of gliadin-specific Th1 cell lines from intestinal biopsies of patients with CD who had transamidated gliadin inhibited IFN-γ expression [10]. In addition, transamidated wheat gliadins evoked a distinct gliadin-specific T cell response in the spleen of DQ8 mice, which was shifted towards a regulatory/anti-inflammatory phenotype [16]. In the present study, we further addressed the issue by dissecting the underlying mechanism at the intestinal level using a previously characterized r-gliadin [13,17]. This protein harbours the most conserved sequence among α-gliadins [13]. Most importantly, we found that nasally administered r-gliadin down-regulated the systemic response to wheat gliadins in DQ8 mice [21], suggesting that this single gliadin may substitute for wheat gliadins to induce tolerance. Herein, the electrophoretic analysis revealed that enzymatic transamidation by mTG generated two discrete bands following Coomassie blue staining and, more distinctively, by Western blotting using an anti-spf serum as probe. We estimated that the lower migrating form represented the quantitatively transamidated r-gliadin, whereas a reduced degree or absence of transamidation characterized the lower mw molecule, co-migrating with untreated r-gliadin. In order to discover peculiarities in its immune reactivity, we nasally immunized DQ8 mice with r-gliadin or transamidated r-gliadin (K-r-gliadin) and tested both molecules in vitro by performing a multiparametric assessment of 12 cytokines. This analysis gave us an overall picture of the immune response generated by both protein forms. By assessing five cytokines associated with innate immunity, we found that both r-gliadin and K-r-gliadin stimulated secretion of macrophage GMCSF and TNF-α with comparable intensity, whereas only MLN cells from mice immunised with r-gliadin produced IL-6. Unexpectedly, very low levels of IL-12 were detected. This is a heterodimeric cytokine produced by different APCs to activate a Th1 response. On the other hand, la Sala et al. [22] reported that the use of CT inhibited the IL-12 related transcription factor IRF8 through up-regulation of intracellular cAMP, providing an explanation to our finding. Nevertheless, the analysis of adaptive immunity showed that sensitised MLN cells mainly secreted IFN-γ with very low levels of IL-10 and undetectable levels of IL-4 and IL-2, indicating that a Th1-like pattern was still induced by both antigens. Notably, r-gliadin-specific intestinal cells produced significantly lower amounts of IFN-γ when challenged with K-r-gliadin, which is in line with previous results in spleen cells tested with transamidated wheat gliadins [10,15,16]. In addition, stimulated MLN cells also produced some levels of IL-5 and IL-13, which are Th2-like cytokines that are mainly associated with B-cell responses. However, differently from IFN-γ, transamidation did not decrease their secretion in r-gliadin-specific T cells, suggesting that the enzymatic modification of r-gliadin specifically targeted the Th1 response. The analysis of MLN cells isolated from mice immunised with K-r-gliadin further confirmed the ability of the transamidation reaction to reduce the intensity of the intestinal response but without changing its phenotype, as lower levels of secretion of IFN-γ, IL-5 and IL-13 were detected following cell stimulation with both antigens. Tandem mass spectrometric analyses of peptides originating from r-gliadin regions 122–136 and 212–238, encompassing immunodominant peptides p13 and p23, confirmed that Q residues deamidated by tTG were also transamidated by mTG [13]. This was in agreement with our previous structural analyses of celiacogenic epitopes, showing that mTG was able to modify the same glutamine residues that were already identified as deamidation sites recognized by tTG [13,14]. However, herein, the transamidation reaction catalysed by mTG yielded a complex mixture of modified forms of K-r-gliadin. In fact, mTG was able to modify other Q residues present in p13 and p23, probably due to its lower site-specificity that leads to extensive transamidation of r-gliadin. Dekking et al. compared the site-specificity of mTG to that of tTG in a deamidation reaction of synthetic gluten peptides [23]. In good agreement with our data, they observed that mTG deamidated those Q residues that were deamidated by tTG. They also found a wider mTG substrate specificity [23]. In silico studies of the interaction between p13 and p23 peptides with DQ8 suggested further interesting observations. Both peptides have been modelled by similarity to those complexed to DQ8 in experimental structures, with the insertion of a gap in the sequence alignment. The insertion of gaps in the alignment is a commonly used procedure to adapt, at the best, the sequences and reach higher similarity in alignment. However, if applied in the modelling of a short segment with anchor requirements, as in our case, the gap could disrupt the expected match of peptide side chains with the anchor positions. Despite the gap in the sequence alignment, we noted that the modelling procedure was able to generate complexes in which the peptide amino acids occupied the expected anchor positions, thus suggesting that P1-P9 binding sites could be covered by peptide segments of nine and also of eight amino acids. Moreover, in our models, we noted that the glutamines, following deamidation, formed salt bridges with side chains positively charged on the surface of DQ8. This interaction was not possible in the case of transamidation of the glutamines, due to the absence of negative charges on the peptide. Furthermore, it was interesting to note that the DQ8 amino acid interacting with the glutamate side chain on the peptide was Arg78 of the alpha chain. This amino acid was in a position that presented differences among the alleles. In particular, the DQ8 alpha chain coded by DQ8A1*03 presented the insertion of an Arg between Arg78 and Phe79, thus resulting in a repetition of two Arg residues. The presence of one Arg in this region of the DQ2 alpha chain could still generate a salt bridge with the glutamate residue in the peptide. On the contrary, it was interesting to observe that alleles DQA1*01:01-07 presented two Gly residues at the same positions, instead of the two Arg residues (see the Natural variant VAR_060510 of the UniProt P01909 entry), thus resulting in the inability to form salt bridges with the deamidated glutamines. This suggested a peculiar interaction with the alpha allele in DQ8, still possible in DQ2, and was not possible for DQ5 and DQ6 (that use allele DQA1*01 for encoding the alpha chain and are present in individuals not affected by CD). This observation also confirmed our previous studies on the interaction between DQ2 and gliadin peptides [24]. We reported that deamidated glutamines might form a negative to positive charge interaction with Lys71 of the DQ2 beta chain. Only the DQ2B1*020x alleles have three positively charged residues in that region (i.e., Arg70-Lys71-Arg72), while in other alleles, the positively charged residues were at least in part substituted by neutral or negatively charged residues [24]. Taken together, our structural findings could well parallel with the specific reduction of the Th1 immune potential of K-r-gliadin. On the other hand, we also detected a strong transamidation-independent secretion of IL-17 following challenge in vitro in both sensitised MLN cells. Furthermore, IL-17 was the only cytokine among the 12 studied that both r-gliadin-specific and K-r-gliadin-specific MLN cells secreted in the presence of wheat gliadins. Th17 cells are the major source of IL-17. Although Th17 cells and Treg cells orchestrate opposite functions, they share common pathways for their differentiation [25]. Additionally, IL-17 may inhibit the development of Th1 cells [25]. The counter-regulatory effects of Th17 on Th1 lineage cytokines may contribute to limit the inhibitory effects of IFN-γ on Th17 development. Most importantly, Th17 cells could totally transdifferentiate into Treg cells [26]. Then, the plasticity of Th17 cells could contribute to the resolution of a phlogistic condition.

## 4. Materials and Methods

### 4.1. Antigens

Gliadin was extracted from wheat flour using a modified Osborne procedure [27]. The recombinant α-gliadin (r-gliadin) (Figure 1A) was produced as described in Rossi et al. [28], except that proteins were precipitated by adding two volumes of chilled 20% trichloroacetic acid to eliminate any residual bacterial contaminant. The pellet was then washed with acetone, air-dried, and stored at −80 °C. For transamidation, r-gliadin was solubilized in 50 mM acetic acid/PBS pH 7.0 (5 mg/mL), containing 20 mM lysine ethyl ester (Sigma-Aldrich, Milan, Italy). Afterwards, 8 U/mg microbial transglutaminase (mTG; ACTIVA^®^WM, specific activity: 81 to 135 U/g, Ajinomoto Foods Hamburg, Hamburg, Germany) was added and the reaction was conducted at 30 °C for 2 h. Protein content was determined by Bradford assay [29], and the protein pattern was qualitatively analysed by 12% SDS-PAGE and Coomassie R-250 blue staining. Preparation of peptic-tryptic digests (pt) of wheat gliadin and r-gliadin was performed as previously described [15].

### 4.2. Mice and Treatments

Transgenic mice expressing the HLA-DQ8 molecule in the absence of endogenous mouse class II genes [30] were reared for several generations on a gluten-free diet (Altromin-MT-mod, Rieper SpA, Bolzano, Italy) in pathogen-free conditions at our animal facility (accreditation n. 164/99-A). For nasal immunization, mice were administered with 20 µg pt r-gliadin or pt transamidated r-gliadin (K-r-gliadin) plus 1 µg cholera toxin (CT) (Sigma-Aldrich, Milan, Italy) in PBS buffer at 10 µL per nostril. Six boosters containing the same amount of antigen and adjuvant were administered at weekly intervals. Mice were sacrificed on day 49 to recover their mesenteric lymph nodes (MLNs).

### 4.3. Western Blotting

Protein aliquots (50 μg) were fractionated by 12% SDS-PAGE, blotted onto Immobilon^TM^ PVDF membranes (Millipore, Billerica, MA, USA) and probed with in-house produced mouse anti-sera toward wheat gliadin (1:10,000 dilution) or transamidated wheat gliadin (spf) (1:10,000 dilution) [14]. After washing, the membranes were incubated with peroxidase-conjugated antibodies against mouse IgG (Dako SpA, Milano, Italy, 1:4000 dilution). Finally, immunodetection was performed using Hyperfilm™ ECL reagent (Amersham-GE Healthcare Europe GmbH, Glattbrugg, Switzerland).

### 4.4. In Vitro Culture of Mesenteric Lymph Node Cells

Mesenteric lymph nodes (MLNs) were passed through a stainless steel wire mesh to dissociate the cells. They were incubated in culture medium (RPMI 1640 containing 10% inactivated FCS, 100 U/mL penicillin, 100 µg/mL streptomycin, 1% non-essential amino acids, 2 mM glutamine, and 50 µM 2-mercaptoethanol) with pt gliadin (200 μg/mL), pt r-gliadin or pt K-r-gliadin (50 μg/mL) for 72 h. The supernatants were then collected and stored at −80 °C.

### 4.5. Cytokine Analysis

Culture supernatants were assayed with a Luminex 200 analyser (Luminex Corporation, Austin, TX, USA) using the cytokine mouse magnetic 20-plex panel (Invitrogen, Life Technologies Corporation, Frederick, MD, USA). The amount of surface fluorescence of the reported dye was related to the cytokine concentration of the sample. Quantitative results were determined from the standard curve using xPONENT^®^ 4.2, a logistic 4-parameter curve fit software (Invitrogen).

### 4.6. Identification of Transamidated Residues in K-r-Gliadin by Tandem Mass Spectrometry

K-r-gliadin was digested with elastase (Sigma-Aldrich) in ammonium bicarbonate 50 mM, pH 8.5 for 2 h at 37 °C using an enzyme to substrate ratio of 1:50 *w*/*w*. The peptide mixture was then reduced in the same buffer, containing a 10:1 molar excess of dithiothreitol over the SH groups for 1 h at 37 °C. Alkylation of cysteine residues was carried out in the same buffer with a 5:1 molar excess of iodoacetamide over the total SH groups for 30 min at room temperature in the dark. The obtained peptide mixture was analysed using a Q-Exactive^TM^ mass spectrometer (Thermo Fisher Scientific, Waltham, MA, USA) interfaced with an UltiMate 3000 RSLCnano LC system (Thermo Fisher Scientific, Waltham, MA, USA). Peptide mixtures were loaded on a pre-column (Acclaim™ PepMap™ 100 C18 HPLC Columns, 0.1 × 20 mm, 5 μm, Thermo Fisher Scientific, Waltham, MA, USA), using 0.05% formic acid and 2% acetonitrile at a flow rate of 2 μL/min. The peptide separation was performed at 40 °C using a C18 column (EASY-Spray™ HPLC Columns, 75 μm × 250 mm, 2 μm, 100 Å, Thermo Fisher Scientific, Waltham, MA, USA), using 0.1% formic acid as eluent A and 80% acetonitrile in 0.08% formic acid as eluent B at a flow rate of 0.3 μL/min and a linear gradient from 2% to 50% B over 40 min, hold for 20 min, from 50% to 90% B over 1 min, hold for 20 min before column re-equilibration to 2% B. Mass spectra were acquired in the *m*/*z* range 350–1600. Data acquisition was performed in a data dependent mode Full MS/ddMS2, enabling the acquisition of fragmentation (MS/MS) spectra for the 10 most intense precursor ions (top 10) and dynamic exclusion of 10 s. Resolution was set to 70,000 for MS spectra acquisition and 17,500 for MS/MS spectra acquisition. MS data processing for identification of the mTG modified Q residues was performed using the ProteomeDiscoverer™ platform (version 2.1.1.21; Thermo Fisher Scientific, Waltham, MA, USA) interfaced with the Sequest HT Search Engine server. The parameters used for data analysis were as follows: protein sequence accession number Q9ZP09 (from the UniProtKB database, previous accession number: aj130948) and a contaminant protein database (PD_Contaminants_2015_5.fasta, provided by the manufacturer), no proteolytic enzyme, carbamidomethyl as fixed modification for cysteine residues, oxidation of methionine residues, pyroglutamic acid for N-terminal glutamine residues, addition of lysine (+129.079 uma) and lysine ethyl ester (+157.1102 uma) as dynamic modification, 20 ppm mass tolerance for precursor ions and 0.02 Da mass tolerance for MS/MS fragments. The transamidation sites identified by the ProteomeDiscoverer™ platform were also confirmed by manual interpretation of the MS/MS spectra.

### 4.7. Molecular Modelling Procedures

Molecular models of the interaction of α-gliadin immunodominant peptides p13 and p23 with HLA-DQ8 (DQ8) have been obtained by comparative modelling procedures. General information about the amino acid sequences of the alpha and beta chains of DQ8 were retrieved by UniProt database [19] (https://www.uniprot.org; accessed on 31 January 2021). In particular, alpha chain has been obtained from the accession number P01909 and beta chain from accession number P01920, respectively. PDB database [31] (https://www.rcsb.org/; accessed on 30 March 2021) has been searched in order to find suitable DQ8-peptide complexes to be used as templates for the modelling procedure. Three out of 16 complexes were selected for their structural features (i.e., best resolution, stereochemical quality by Ramachandran plot, Q-mean, Z-score by PROSAWeb, lowest number of missing atoms or missing residues). Each structure was incomplete for missing portions, both at internal and terminal regions. Therefore, in order to obtain a model of DQ8 without missing internal regions, the structure of DQ8 was modelled using the same alpha and beta chain sequences of PDB 2NNA by comparative modelling to the template structures of 2NNA (A and B chains), 1JK8 (A and B chains), and 6DFX (A and B chains). The structures of p13 and p23 in complex with DQ8 have been modelled on the basis of three different alignments of each peptide sequence to template peptide sequences available in the PDB structures. The different alignments (see Results) have been applied to verify the best fitting of peptide amino acids in the anchor positions described for the gluten peptide interaction with DQ8 [32]. For models 1 and 2, the peptide sequences have been aligned with the sequence of the gluten peptide from 2NNA (chain C, 13 residues), insulin peptide from 1JK8 (chain C, 14 residues), p8E9E peptide from 6DFX (chain B, 19 residues), and glia gamma1 peptide from 5KSA (chain J, 11 residues). In model 3, in addition to the already listed peptides, we also used peptides from 3I78 (chain A) for peptide p13 and from 5I35 (chain A) to model the tails outside the DQ8 binding pocket. Furthermore, as the alignment to generate model 3 included a gap into the peptide sequence, an iterative modelling procedure was performed to find the best position of the gap and to refine the structure. Modeller 9.19 [33] was used to build 10 structures for each model of interaction, which were assessed for their quality by means of different tools according to standard procedures used in our previous protein modelling studies [24,34], and the best structure was finally selected as the model of interaction. In more detail, Z-score was evaluated by ProSA-web (https://prosa.services.came.sbg.ac.at/prosa.php; accessed on 30 March 2021). Ramachandran plots were created by PROCHECK (https://saves.mbi.ucla.edu/; accessed on 30 March 2021). Q-mean was obtained by QMEAN SWISS-MODEL (https://swissmodel.expasy.org/qmean/; accessed on 30 March 2021). Moreover, PDBePISA (http://www.ebi.ac.uk/pdbe/prot_int/pistart.html; accessed on 30 March 2021) was used to evaluate the intrachain solvation free energy gain upon formation of the interfaces with its relative *p*-value, residues at the interfaces between chains and their potential involvement in H-bond or salt bridge formation [35]. The PyMOL Molecular Graphics System, Version 1.3, Schrödinger, LLC, was used to visualize and compare the models that were obtained.

### 4.8. Statistical Analysis

The immunological results were evaluated by one-way analysis of variance (ANOVA) and Tukey test post hoc analysis. For all tests, *p* < 0.05 was selected as the level denoting a statistically significant difference.

## 5. Conclusions

Our data pointed out that transamidated r-gliadin did not evoke a different repertoire of T cells recognizing the immunodominant peptides of r-gliadin. The main output was that K-r-gliadin drove induction of intestinal IL-17 in the absence of IL-6 or by downregulating pro-inflammatory cytokines, such as IFN-γ, probably as a consequence of a reduced binding affinity for DQ8. The peculiarity of this cytokine profile, along with the intrinsic plasticity of Th17 cells, might then be considered in the perspective to establish an innovative prophylactic approach that is based on the use of transamidated gliadin for boosting mucosal tolerance to wheat gliadins.

## Data Availability

The data presented in this study are available upon reasonable request from the corresponding author.

## Supplementary Materials

The following are available online at https://www.mdpi.com/article/10.3390/ijms22137019/s1. Figure S1: Identification of Q residues transamidated by mTG. MS/MS spectrum of the peptide MDVVLQQH (peptide 129–136 of the K-r-gliadin) containing two modified Q residues (precursor ion at *m*/*z* 415.218). The table included in the figure reports the theoretical b and y fragment ions for the fully modified peptide. Signals present in the MS/MS spectrum matched to b ions (highlighted in red) and y ions (highlighted in blue). K-Q refers to transamidated glutamine residues. Figure S2: Identification of Q residues transamidated by mTG. MS/MS spectrum of the peptide SFRPSQQNPQAQGSVQPQ (peptide 212–229 of the K-r-gliadin) containing three modified Q residues (precursor ion at *m*/*z* 593.558). The table included in the figure reports the theoretical b and y fragment ions for the fully modified peptide. Signals present in the MS/MS spectrum matched to b ions (highlighted in red) and y ions (highlighted in blue). K-Q refers to transamidated glutamine residues. Figure S3: Identification of Q residues transamidated by mTG. MS/MS spectrum of the peptide QLPQFEEIR (peptide 230–238 of the K-r-gliadin) containing two modified Q residues (precursor ion at *m*/*z* 473.263). The table included in the figure reports the theoretical b and y fragment ions for the fully modified peptide. Signals present in the MS/MS spectrum matched to b ions (highlighted in red) and y ions (highlighted in blue). K-Q refers to transamidated glutamine residues.
