# Supplementary figures and images for: Transamidation Down-Regulates Intestinal Immunity of Recombinant α-Gliadin in HLA-DQ8 Transgenic Mice
Source: Int J Mol Sci. 2021 Jun 29;22(13):7019. doi: 10.3390/ijms22137019 (PMC8268696; doi:10.3390/ijms22137019)

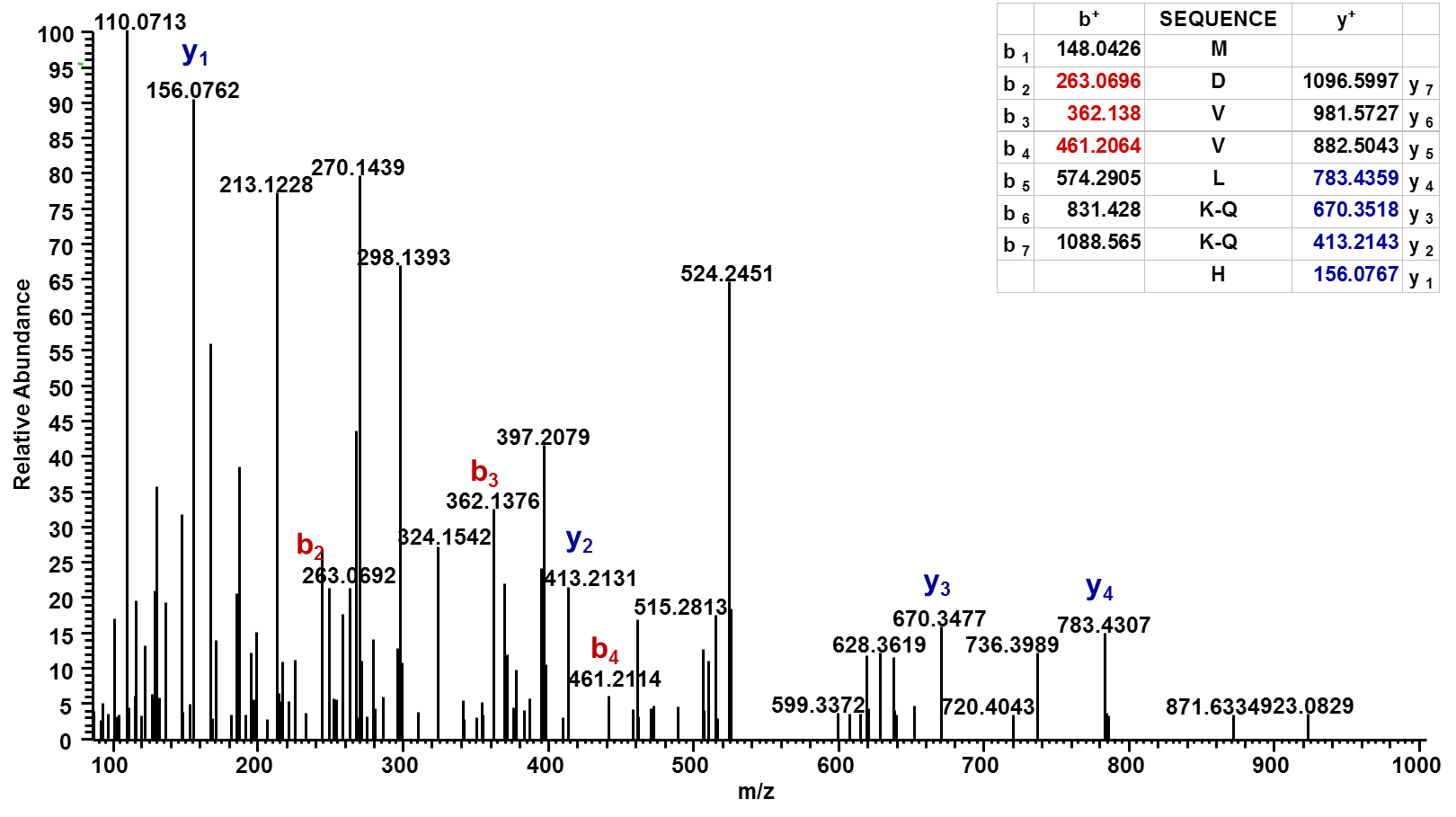

Supplement: Supplementary file 1 [file ijms-22-07019-s001.zip › Supplemetary Figure S1.jpg]

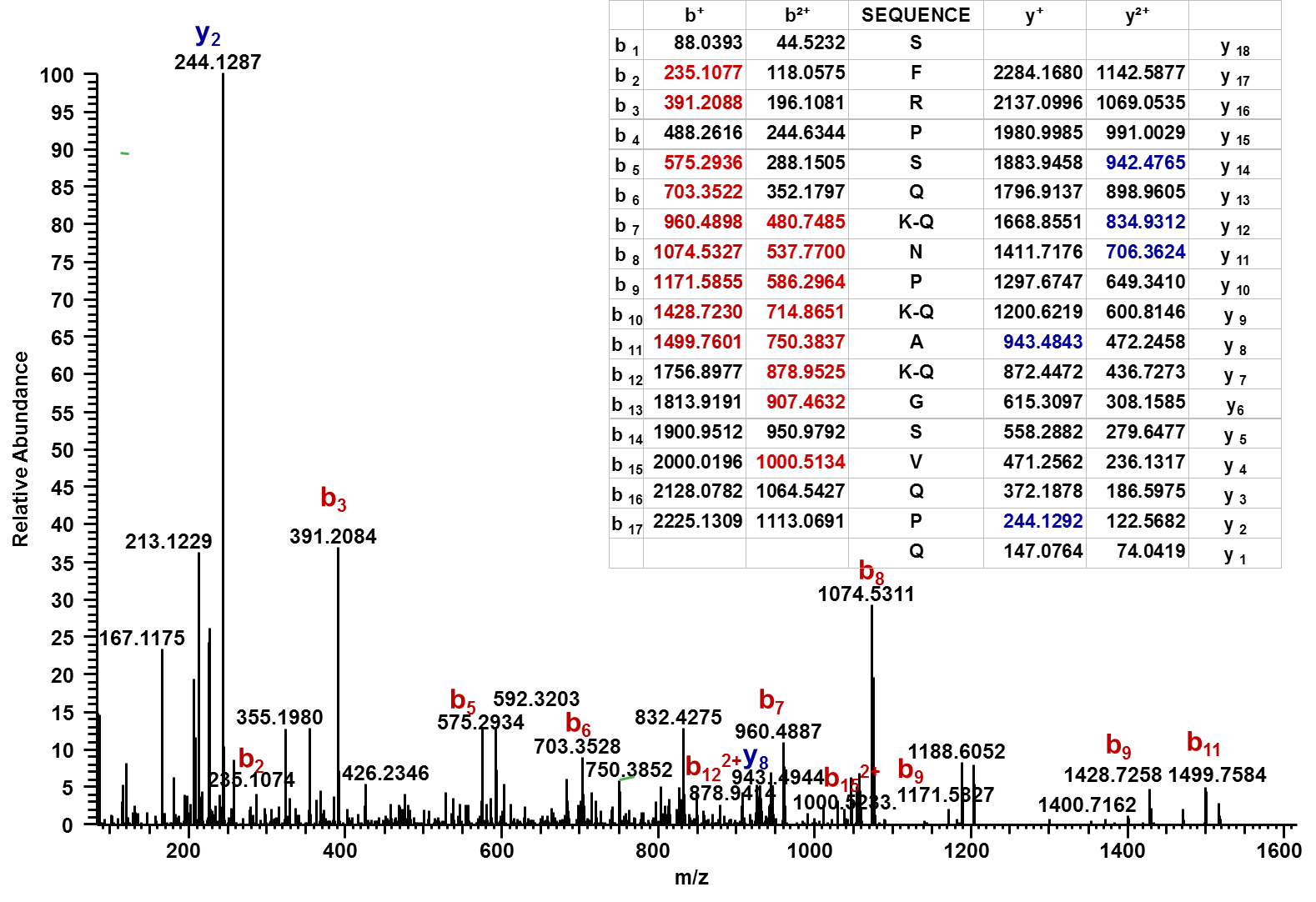

Supplement: Supplementary file 1 [file ijms-22-07019-s001.zip › Supplemetary Figure S2.jpg]

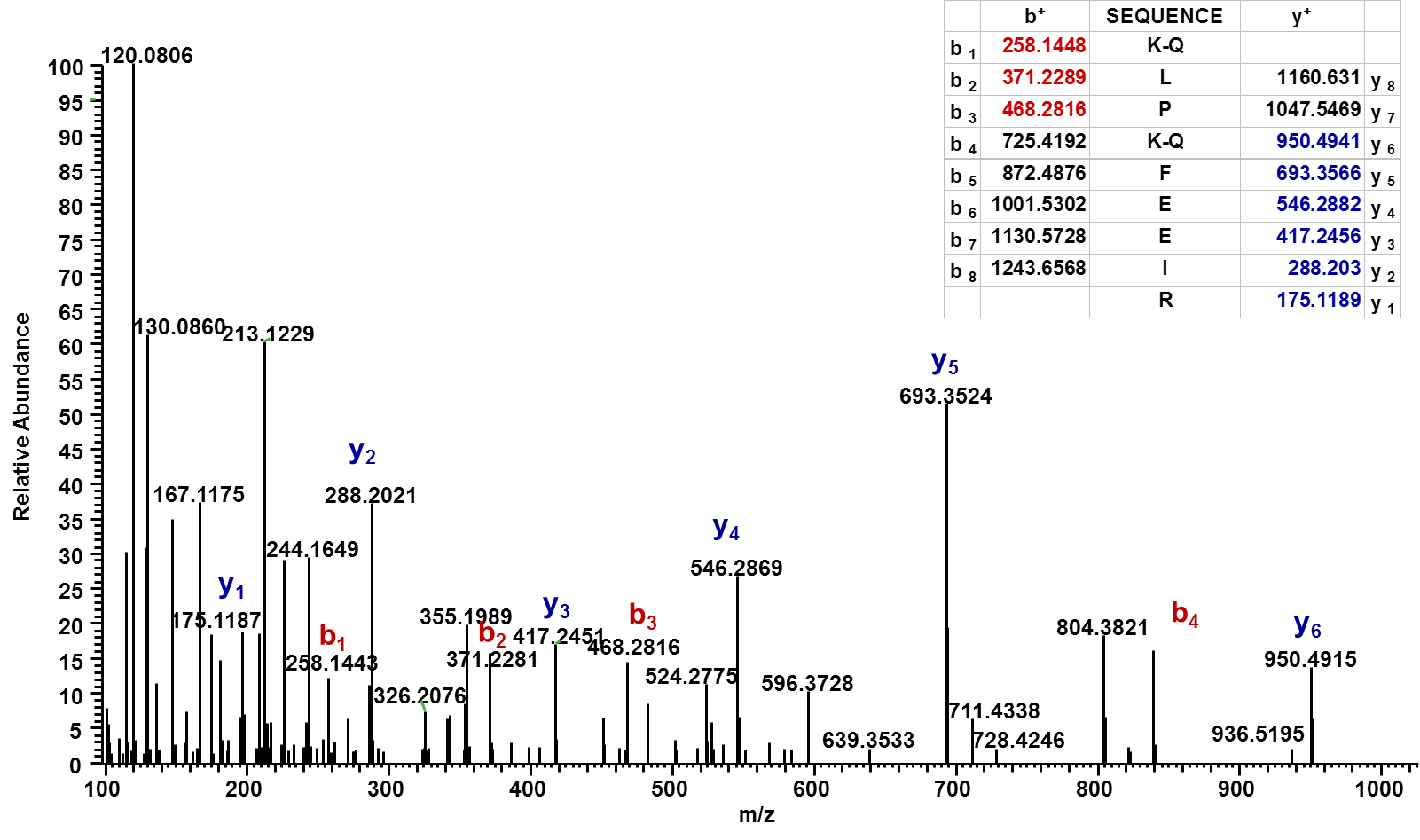

Supplement: Supplementary file 1 [file ijms-22-07019-s001.zip › Supplemetary Figure S3.jpg]
